# American Academy of Dental Science

**Published:** 1889-03

**Authors:** V. C. Pond

**Affiliations:** Recording Secretary


### Reports of Society Meetings.

### AMERICAN ACADEMY OF DENTAL SCIENCE.

The January meeting was held with the president, Dr. C. P. Wilson, in the chair.

The evening was devoted to the exhibition and explanation of new instruments and methods.

*Dr. Clapp* showed specimens of combination fillings of gold and amalgam as described in his paper published in the December *Cosmos.*

*Dr. Fillebrown* explained the use of McLean disks for sharpening, as described by Dr. McLean at the union meeting of the Connecticut Valley and Massachusetts Dental Societies, and reported in the December number of the *Independent Practitioner.*

*Dr. Pond* exhibited a set of forceps and read the following paper:

Mr. President and Members of the Academy—I am aware that extracting is not the important subject it once was, and that forceps do not receive as much attention as formerly; but as it is still sometimes necessary to extract teeth, and as the operation is many times attended with difficulties to the operator as well as to the patient, I should like to bring to your attention a set of forceps, which I think have some advantages over those in general use.

They are the results of many experiments, and while there are no very noticable changes in appearance, yet they differ radically from those usually sold.

There are ten pairs in all, and they are designed to extract all the teeth. There are but two patterns of handles. All the forceps for the upper teeth have straight handles; those for the lower teeth have the familiar curve adopted by most makers. The ends of the handles of the upper forceps are rolled over and rounded, forming a large, smooth surface to rest in the palm of the hand when in use.

In extracting they are intended to be held with the hand in front and above the handle; the rounded end of one handle in the palm of the hand and the other handle between the second and third fingers.

This position allows the operator to use all necessary force in pushing the beaks upward without allowing the forceps to slip through the hand. It also allows the wrist to be kept straight and the operator to stand in such a position that he can watch the tooth, even when exerting all his strength. In holding forceps with the hand back of and under the handle, the tendency is for the forceps to slip through the hand on the least upward pressure, and the wrist is twisted into a position unfavorable to the use of force.

The wrist is also twisted when forceps with curved handles are used for extraction of upper teeth, and no doubt all of you have seen operators in using such forceps forced, when they applied much power, to straighten up, holding the patient's head between the arm and body, and thereby assuming such a position that it was absolutely impossible for them to see what they were doing.

The lower forceps are held with the thumb above and just back of the joint, and partially between the handles. Great downward pressure can be exerted when necessary; the thumb will assist in keeping the forceps open, and as the handles at this point are slightly rounded, there will be none of that disagreeable pinching of the thumb, when the beaks are closed, which doubtless you have all experienced. The joints of all the forceps are rounded. I consider this form much better than the square or octagon joint, as it is smoother to the lips and much less liable to nip the soft parts. It is also fully as strong, and being much smaller, a better chance is allowed to see into the mouth.

Great care has been taken to have the pins in the joints in the right positions, and they are much nearer the beaks in these forceps than in the older patterns. You will find many forceps with the fulcrum three inches from the beaks, and they will grasp a tooth with about as much force as you can with your fingers. Such forceps, unless you close the handles with great force, will rock on the tooth and surely break the crown, and the use of much power in keeping the handles closed will interfere with extracting. Besides, with the fulcrum at a great distance from the beaks, it is necessary to lengthen the handles considerably to gain power, and the hand is too far from the tooth, which is another disadvantage.

In forceps of English make, the pins are much nearer the beaks than in this set. They have great power; but my experience has led me to believe that the leverage is too great. As an English dentist once expressed it, they certainly extract a great many crowns.

One of the greatest improvements in these forceps is in the shapes of the beaks. They will fit the teeth for which they are intended. Forceps may be made to crush crowns like a nutcracker, or to cut them off like cutting pliers, and many in the dental depots are constructed on one or the other of these principles.

You can go into any dental depot to-day, and find forceps intended for the upper molars with the beaks parallel, and you all know how seldom you find an upper molar with the palatal and buccal walls parallel. Such things show conclusively how little care is exercised by the buyer or seller, and is certainly evidence of great carelessness on the part of dentists.

One of the largest forcep makers in this country said in regard to this matter: "I know better than to make forceps as I do; but the managers and clerks of the depots don't know, and accept anything that is well finished, and some one buys them; and as I am paid by the piece, I can make more money by making them in this way." Not a very creditable statement to be made for the maker; but one that shows his opinion of the skill and knowledge exercised by us in the matter.

The shapes of the beaks of these forceps were determined by filling the blanks with wax, and closing them upon the crowns of the teeth for which they were intended. Repeated trials gave a typical shape, to which the beaks were cut.

There are no serrations in the beaks, as they are practically useless, and weaken the beaks to such an extent that it is necessary to make them much thicker than when no serrations are cut.

You will also notice that these beaks are thinned just at the tips, to allow them to slip under the gums without cutting. The upper molar forceps may look somewhat one-sided, but they fit the teeth; and the forceps for the upper front teeth have one beak (the outer) wider than the other, to conform to the shape of those teeth, which is quite an advantage, and a point that was entirely overlooked by most makers, and, in fact, is now. The forcep for the lower third molar has a long, small joint and small beak, which enables the operator to reach that tooth when there is very little room, and with it you can succeed many times when it is impossible to reach the tooth with ordinary forceps.

Many of the points to which I have called your attention are trifling, I know; but as attention to trifles makes perfection, so attention to details is necessary to produce good forceps.

At the present time there is great carelessness or ignorance shown in the manufacture of forceps, which, perhaps, is to be expected, and that which directly concerns us, carelessness in buying and using. Many students buy their forceps before they have any idea of extracting, and continue to use the same set in practice. No operator is so skillful that he does not need good instruments. His great skill may enable him to succeed with poor instruments, but he would attain better results with good ones. Poor forceps are responsible for much of the poor extracting, and poor extracting is by no means uncommon in spite of the advance of our specialty.

Anything that is worth doing is worth doing well, and if we are going to extract, let us know what points we wish embodied in our forceps and see that we have them. Be more careful and exacting in what we buy, and the makers will be forced to improve. Do not depend upon them, and take anything they recommend and assure us is just right. They do not and should not know what is best. Most of them know nothing whatever about extracting; and although some claim to have competent dentists engaged to examine all of their products, and on that ground almost refuse suggestions, the results do not show evidence of great thought or knowledge.

The dentist should be the judge, and should exercise his judgment in such a way that poor extracting, due to poor forceps, should be a thing of the past; and many of the patterns of forceps which now have a large sale would find their proper place in the old junk, much to the relief of suffering humanity.

*Dr. Ham*—I like Dr. Pond's forceps very much, and can suggest one thing which I think would be an improvement, that is to have the sharp edges of the joints beveled. I formerly had trouble, as I presume many of you may have had, with forceps cutting the lips when extracting, and I remedied it by beveling the joints of my forceps. I hear the suggestion that a napkin can be used over the lips; but that is open to the same objection as large joints—it obstructs the view.

*Dr. Loveland*—I have here a gum-cutter, which I have designed to use in cases of difficult or painful eruption of the third molar. It is not a perfect instrument, and is exhibited in the hope that some one may take the ideas and develop them. I find difficulty in using the rotary cutters in the back of the mouth, and the guillotine will not cut through thick or tough tissues.

This cutter resembles a pair of forceps or pliers with the ends of the jaws rounded. The lower one smooth and thin, to slip under the piece of gum to be removed; and the upper one thicker, and having fastened around its outer edge a very thin, steel knife. When the handles are closed the knife on the upper jaw is forced against the smooth lower jaw in the same manner as in a punch for perforating leather. It works very nicely, and will remove a piece of the gum with a smooth, clean cut.

*Dr. Wilson*—Gentlemen: I desire to show a new saw frame which I have designed. The long handle, covered with hard rubber, makes it easy to manipulate; then, again, different lengths of saws can be used. When the saws are adjusted, and the screw at the end of the handle tightened, it is impossible for them to move. I will pass around different saws I used with this frame. You will notice they are of different lengths and thicknesses. The very thin, paper-like saws are very useful in trimming down cement filling between teeth that are close together.

I also desire to show a new mirror frame. This frame is somewhat similar to other split frame mirrors, with the exception of its being much lighter and less bulky. The screw that joins the handle to the frame proper is high up in the handle, where there is plenty of room for it. In setting a new glass I place an instrument in the yoke of the frame, and spring it open sufficiently to let the mirror drop out.

*Dr. Hopkins*—I should like to call your attention to a napkin holder patterned after one made by the late Dr. Tucker. It is an ancient appliance worthy of being modernized, and is almost indispensible in those difficult cases beyond the use of the rubber dam. It is made of German silver wire, so shaped as to include all the teeth on one side of the lower jaw in a way to closely press the folded napkin against the gums excluding the saliva, and is held in position by a handle in the hands of the patient.

*Dr. Codman*—I have had some cases recently where it was desirable to keep a tooth dry for some time after filling and have devised the following method. By its use you can do away with the disagreeable mouthful of rubber dam, and allow your patient to sit comfortably in a chair and read, while you attend to others; or you can operate on other teeth in the same mouth.

The rubber dam is applied and tied as usual; after finishing the filling or whatever is to be done, the dam is gathered over the crown of the tooth and tied tightly with a ligature as close to the tooth as possible. The dam above the ligature is cut away and you have your tooth in a tight rubber bag and can keep it there as long as you wish, with very little discomfort to your patient.

*Dr. Preston*—I have a method of extracting badly decayed roots, difficult to grasp with forceps, which I will endeavor to explain. With the engine drill a hole in the root as for a pivot. Take a common wood screw, such as carpenters use, of a proper size and with a file flatten it on four sides making a tap. With this tap cut a thread in the root; then take a similar screw, not flattened, and screw it into the thread cut into the root with a small screw-driver or pliers. Extract the root by taking hold of the screw with forceps. It is not necessary to cut the gums, and the worst roots can be removed without even scratching the surrounding tissue.

*Dr. Fillebrown*—I have lately been using creolin as a substitute for carbolic acid, for washing instruments and for general disinfecting purposes, and as a surgical dressing. I like it very much, as it is free from the disagreeable odor of the acid and is not poisonous. I am now using creolin gauze as a dressing for an antrum upon which I operated lately, curetting a large patch of pyogenic membrane from the under surface of the infra-orbital plate. It keeps the wound perfectly antiseptic and free from odor. It is used extensively throughout Continental Europe.

Creolin is a by-product in the manufacture of carbolic acid. It is produced by the addition of resin and soda to the creasote after removal from the latter of all carbolic acid.

It forms a dark-brown, syrupy fluid, with a peculiar odor, somewhat resembling tar. Its taste is at first aromatic, and at last burning.

It is readily soluble in alcohol and ether. With water it is insoluble, but is suspended in the form of emulsion, producing an opaque milky appearance. The most complete emulsion possible is two and one-half per cent.

Creolin belongs to the most powerful antiseptics and disinfectants. As a deodorizer it is unsurpassable. It has hæmostatic properties and promotes granulations. It is non-irritating, and internally is non-poisonous. It is used in form of gauze, ointment, soap or emulsion.

Subject passed. V. C. Pond, *Recording Secretary.*

